# A dense SNP genetic map constructed using restriction site-associated DNA sequencing enables detection of QTLs controlling apple fruit quality

**DOI:** 10.1186/s12864-015-1946-x

**Published:** 2015-10-05

**Authors:** Rui Sun, Yuansheng Chang, Fengqiu Yang, Yi Wang, Hui Li, Yongbo Zhao, Dongmei Chen, Ting Wu, Xinzhong Zhang, Zhenhai Han

**Affiliations:** Institute for Horticultural Plants, College of Agronomy and Biotechnology, China Agricultural University, Beijing, 100193 China; Changli Institute for Pomology, Hebei Academy of Agricultural and Forestry Science, Changli, Heibei 066600 China

**Keywords:** Apple, Candidate genes, Fruit quality traits, Genetic maps, QTL, RADseq, SNP

## Abstract

**Background:**

Genetic map based quantitative trait locus (QTL) analysis is an important method for studying important horticultural traits in apple. To facilitate molecular breeding studies of fruit quality traits in apple, we aim to construct a high density map which was efficient for QTL mapping and possible to search for candidate genes directly in mapped QTLs regions.

**Methods:**

A total of 1733 F1 seedlings derived from ‘Jonathan’ × ‘Golden Delicious’ was used for the map constructionand QTL analysis. The SNP markers were developed by restriction site-associated DNA sequencing (RADseq). Phenotyping data of fruit quality traits were calculated in 2008-2011. Once QTLs were mapped, candidate genes were searched for in the corresponding regions of the apple genome sequence underlying the QTLs. Then some of the candidate genes were validated using real-time PCR.

**Results:**

A high-density genetic map with 3441 SNP markers from 297 individuals was generated. Of the 3441 markers, 2017 were mapped to ‘Jonathan’ with a length of 1343.4 cM and the average distance between markers was 0.67 cM, 1932 were mapped to ‘Golden Delicious’ with a length of 1516.0 cM and the average distance between markers was 0.78 cM. Twelve significant QTLs linked to the control of fruit weight, fruit firmness, sugar content and fruit acidity were mapped to seven linkage groups. Based on gene annotation, 80, 64 and 17 genes related to fruit weight, fruit firmness and fruit acidity, respectively, were analyzed.Among the 17 candidate genes associated with control of fruit acidity, changes in the expression of MDP0000582174 (MdMYB4) were in agreement with the pattern of changes in malic acid content in apple during ripening, and the relative expression of MDP0000239624 (MdME) was significantly correlated withfruit acidity.

**Conclusions:**

We demonstrated the construction of a dense SNP genetic map in apple using next generation sequencing and that the increased resolution enabled the detection of narrow interval QTLs linked to the three fruit quality traits assessed. The candidate genes MDP0000582174 and MDP0000239624 were found to be related to fruit acidity regulation. We conclude that application of RADseq for genetic map construction improved the precision of QTL detection and should be utilized in future studies on the regulatory mechanisms of important fruit traits in apple.

**Electronic supplementary material:**

The online version of this article (doi:10.1186/s12864-015-1946-x) contains supplementary material, which is available to authorized users.

## Background

Marker assisted selection (MAS) is an efficient approach for plant breeding, especially for woody perennials such as apple. Generally, MAS is dependent on high-density genetic linkage maps. Before 2010, a number of genetic maps were constructed using different types of markers, such as random amplified polymorphic DNAs (RAPDs), amplified fragment length polymorphisms (AFLPs), sequence characterized amplified regions (SCARs), and simple sequence repeats (SSRs). A saturated reference map for apple using 840 molecular markers (475 AFLPs, 235 RAPDs, 129 SSRs, and 1 SCAR) was published in 2003 [1]. Genetic maps of ‘Braeburn’ and ‘Telamon’ were constructed in 2005 using 257 F1 mapping populations. The maps of ‘Braeburn’ consisted of 245 AFLPs and 19 SSRs, while the ‘Telamon’ maps comprised 242 AFLPs and 17 SSRs [2]. Genetic maps of apple rootstock ‘M9’ × ‘R5’ have been produced containing 316 newly developed SSR marker loci [3].

The whole genome sequence of apple was released in 2010, making the development of massive SNPs and the construction of high-density genetic maps of apple possible [4]. For example, an SNP-based linkage map constructed using M432 *Malus* mapping population comprised 2272 SNP markers, 306 SSR markers, and the S-loci, the average distance between markers was increased to 0.5 cM [5]. The consensus map of ‘Honeycrisp’ was developed using three F1 populations via an SNP chip containing 1091 SNP markers with an average distance of 1.36 cM between consecutive markers [6].

There was no doubt that these high-density maps could be used in breeding programs more efficiently; however, QTL analysis mostly relied on polymorphisms between the parents of mapping populations. Many markers have different genotypes or linkage relationships among different hybrid populations, therefore it is not easy to transfer the QTL mapping results acquired from one population to another [7–9]. Thus, there is the need to develop new high-density and standard genetic linkage maps of apple for further research.

SNPs are widely distributed throughout the whole genomes. In pear, 3402159 SNPs were detected, comprising approximately 1.02 % of the whole genome [10]. SNPs were more frequently detected in grape, reaching 7 % [11], and the amount of SNPs obtained in citrus was 1.06million [12]. In apple, the density of SNPs was about 4.4 per kb [4]. Except whole genome sequencing, next-generation sequencing (NGS) was also a strategy for exploring SNPs [13]. RADseq, one of the NGS technologies, was first described by Miller et al. in 2007 [14]. Compared with SNP arrays, RADseq was more rapid, cost effective, and independent of any genome information [15–17]. Recently, several genetic linkage maps were constructed via RADseq and were used for genetic mapping and population evolution in different species, such as pear [18], barley [19], and *Lupinus angustifolius* L. [20].

In this decade, apple consumers care about fruit quality traits, both the appearance and flavor, more than ever. Improving quality traits has been an endless pursuit for apple breeders, and most efforts on QTL mapping have been made on fruit size, firmness, sugar contents, and acidity [21–23].

The involvement of major gene segregation was previously proposed to explain the variations in the fruit acidity of several hybrid populations [24–26]. A major gene controlling fruit acidity in apple, the *Ma* locus, was first mapped to LG16 [27]. In the population ‘Fiesta’ × ‘Discovery’ and ‘Telamon’ × ‘Braeburn’, QTLs of fruit acidity were also mapped to LG16 [21, 22], which confirmed the previous results. Recently, the *Ma* region located on LG16 has been delimited within 150 kb in the Golden Delicious genome [26]. Although there are a few sequence gaps, it is a big step for breeders in uncovering the candidate gene(s). Another major QTL associated with fruit acidity was, however, detected on LG08 [21–23, 28], which is worthy of future research.

The inheritance of fruit sugar contents has been proven to be quantitative [24, 29]. QTLs for sugar content in apple are dispersed on different linkage groups in different hybrid populations. In ‘Fiesta’ × ‘Discovery’, five QTLs for sugar content were mapped to LG03, 06, 08, 09, and 14 using integrated maps, however, in ‘Telamon’ × ‘Braeburn’, two QTLs linked to total soluble solids were located on LG02 and 10 in both parents [21, 22]. In the USDA-NIFA funded RosBREED project, QTLs linked to sugars were detected on apple chromosomes 01, 02, and 16 using almost 1000 individuals [30].

Fruit size and fruit weight are typical quantitative traits, and QTLs linked to these traits always have low rates for the explanation of variance. In ‘Fiesta’ × ‘Discovery’, QTLs associated with fruit weight were detected on eight linkage groups, and the highest explanation of variance among these QTLs was 13 % [21]. In ‘Telamon’ × ‘Braeburn’, fruit weight was attributed to genomic regions dispersed on five linkage groups, LG02, 06, 09, 10, and 17 [22]. However, only LG06 and LG10 were common to the two hybrid populations. Later, by combining the data for fruit weight from two mapping populations ‘Royal Gala’ × ‘Braeburn’ and ‘Starkrimson’ × ‘Granny Smith’, the QTLs were detected on eight linkage groups [31].

Although fruit firmness is also quantitatively inherited, the identification of QTLs on chromosome 10 leads to the discovery of a candidate gene, Md-PG1. The QTL cluster on LG10 associated with the fruit texture parameters was co-located with the Md-PG1_SSR_ marker developed from the sequence 3 kb upstream of the Md-PG1 start codon [32]. It is suggested that Md-PG1 plays an important role impacting fruit firmness in 77 apple cultivars [33].

Our goal in this study was to increase understanding of the genetic control of fruit quality traits in apple, utilizing QTL mapping of several traits associated with quality and subsequent identification of candidate genes, that might provide new insights into the regulation of fruit quality. Although QTL analysis has been a hotspot in apple quality trait research to date, the QTLs detected previously were always within long genomic regions, which are difficult to use in breeding programs and further research. In this work, we set out to remedy this problem by constructing a high-quality genetic map using a RADseq strategy, which we could use as the basis for QTL mapping and candidate gene analysis.

## Methods

### Plant materials

The hybrid population, including 1733 seedlings used in this experiment, was generated from the cross between ‘Jonathan’ as the maternal parent and ‘Golden Delicious’ as the pollen parent in 2002. The seedlings were all grown on their own roots and planted at a density of 0.5 m × 2 m at the Changli Institute of Pomology (Hebei, China). The orchard was subjected to conventional field managements and pest control. At the first apple harvest in 2008, the plants were 6 years old.

### Development of SNP markers using RADseq

From the segregation population, 318 individuals were selected randomly to construct the genetic map. Genomic DNA of the 318 F1 individuals and two parents, ‘Jonathan’ and ‘Golden Delicious’, was extracted from young leaves using a Genomic DNA Isolation Kit (TianGen, Beijing, China) and then processed into RADseq libraries, following the protocol described by Baird and the colleagues [15]. Briefly, the genomic DNA was digested with two restriction endonuleases, *Eco*RI and *Hin*dIII, respectively. P1 adapter, a modified Solexa© adapter (2006 Illumina, Inc., all rights reserved), was ligated to the samples. After P1 adapter ligation, samples were pooled and randomly sheared with a Bioruptor (Diagenode, Belgium), and DNA fragments of the desired length, approximately 500 bp, were gel purified. To complete the construction of DNA libraries, P2 adapter, a divergent modified Solexa© adapter (2006 Illumina, Inc., all rights reserved), was ligated to the obtained DNA fragments at 18 °C. Then the purified and quantified samples were used for PCR amplification. Finally, samples were gel purified again, isolated DNA fragments 300 to 650 bp, and diluted to 1 nM. The prepared DNA libraries were sequenced on an Illumina Hiseq2000 platform at BGI-Shenzhen (Shenzhen, Guangdong, China), using the PE100 (paired-end, 100 bp) strategy. Sequence data were analyzed using custom Perl scripts designed by BGI-Shenzhen (Shenzhen, Guangdong, China) which were reported before [34]. RAD markers were developed using the clean data after removing the adapters, index sequences, and low-quality reads. Reads from each individual were clustered into tag reads by sequence similarity (allowing five mismatches, at most, between any two reads within each tag reads cluster) and clusters with < 3 or > 100 reads were discarded. All the SNPs had total support reads ≥ 5, and for heterozygous SNPs the inferior base depth was ≥ 3. Based on the genotypes of the parents, monomorphic markers were removed. Finally, three types of markers were obtained: lm × ll, nn × np and hk × hk.

### Map construction and marker nomenclature

Markers used for map construction were filtered using the following criteria: a) for each marker, individuals that lacked genotyping data were less than 60 (~20 % of the total); and b) all markers were tested by Chi-square test (p < 0.01). The segregation ratio of lm × ll and nn × np markers was expected to be 1:1, while that of the hk × hk markers was 1:2:1. Genetic linkage maps were generated using JoinMap version 4.1 [35]. A logarithm of the odds (LOD) score of 6.0 was set to distinguish linkage groups. The regression mapping was used as the mapping algorithm, and the genetic distances were calculated based on Kosambi’s mapping function. Both parental and consensus maps were constructed, and markers that appeared in the ‘suspect linkage’ table and individuals carrying several double recombination events were removed.

All mapped markers were named after aligning to the reference genome sequence of apple. The new names include three or four alphabet characters. The first character, ‘h/e’, indicates restriction enzyme, ‘h’ represents *Hin*dIII and ‘e’ represents *Eco*RI. The second letter, ‘m/u/N’, indicates the alignment to the reference genome sequence, ‘m’ represents multiple alignments, ‘u’ represents unique alignment and ‘N’ represents no alignment results. The following letter(s), ‘C’ or ‘M’ or ‘LG’, show(s) the linkage groups numbered in accordance with the chromosome numbers of the genome data base. When the chromosome number of a SNP-containing sequence was the same as the number of linkage groups that the SNP was mapped to, the third alphabet character of the marker name will be ‘C’, eg. huC02.30210127, where ‘C’ is the abbreviation for chromosome. The two-digit number prior to the period represents the chromosome number, and the numerals following the period indicate the exact physical position of the SNP on the chromosome of the apple genome database (C-type markers). If the linkage group number of the mapped SNP did not correspond to the chromosome number in the genome, the characters following the second letter are formatted as LGN_numerals, such as euLG02_006 and hmLG09_605, where ‘LG’ is the abbreviation for linkage group. The following ‘N’, a two-digit number, represents the linkage group number and the numerals following the underscore ‘_’ indicate the number given serially within the linkage group (LG-type markers). Therefore, when the second letter in the nomination is ‘N’, the name of that marker will uniquely follow this format, eg. hNLG02_012 (N-type markers). In the case where an SNP-containing sequence aligned with an unanchored contig in the genome database, the characters after the second letter in the nomination are MX.numerals, such as huM018217.288.21412, where ‘M’ means chromosome 0. The ‘X’ consists of two numerals flanking the first period, representing the contig code in the apple genome database. The numerals following the second period indicate the exact position of the SNP on that contig (M-type markers).

### Phenotyping

Because of the alternate bearing of the segregating population, fruit from 1170, 952, 527, and 106 seedlings were harvested in 2008, 2009, 2010, and 2011, respectively. Ripening apples were harvested from each fruit-bearing individual and six apples from each plant were used for phenotyping of fruit quality traits. Average fruit weight was measured using an electronic balance and recorded in grams for 4 successive years. Fruit firmness was determined on four directions of each fruit using a DESIK-GY-4 penetrometer (Zhejiang Top Instrument Co., Ltd) in 2010 and 2011. Contents of fruit soluble sugar (fructose, glucose, and sucrose) and organic acids (malic, citric, tartaric, oxalic, acetic, and succinic) were analyzed by high-performance liquid chromatography (HPLC) in 2011 [36]. Samples were prepared by grinding 5 g fresh fruit flesh from a mixture of six apples in 10 mL redistilled water. The homogenate was centrifuged at 12,000 rpm for 10 min at room temperature after 30 min in 75 °C water. The pomace was extracted again with 8 mL redistilled water. Then, the combined supernatant was diluted up to 25 mL and filtered through 0.45 μm Millipore filter. The resultant supernatant was used for HPLC analysis. Sugar analyses were performed on a LC-10ATvp chromatograph (Shimadzu Corporation., Kyoto, Japan) with an Asahipak NH2P-50 4E column (4.6 mm × 250 mm) (Showa Denko, Japan) attached to a RID-10A refractive index detector (Shimadzu Corporation, Kyoto, Japan). The mobile phase was acetonitrile/water (73:17), with a flow rate of 1 mL min^−1^ at 40 °C. Organic acids analyses were carried out at 30 °C at a flow rate of 0.5 mL min^−1^ with 0.01 M K_2_HPO_4_ · 3H_2_O, pH 2.6 as the mobile phase using a Waters 600 chromatograph, and a Waters 2487 ultraviolet detector (Waters, Milford, MA, USA). The column used here was a reversed-phase C18 column (4.6 mm × 150 mm) (Sigma Aldrich, St. Louis, MO, USA). The distributions of traits analyses were carried out using the Shapiro-Wilk test.

### QTL analysis

QTL analysis was performed using MapQTL version 6.0 [37] software based on the parental maps and phenotype data from 163, 102, 97, and 80 seedlings in 2008, 2009, 2010, and 2011, respectively. QTLs were detected using interval mapping initially, and the mapping algorithm was a mixed model. Then multiple QTL mapping (MQM) was performed to detect additional QTLs that might be masked by the major QTLs. After a 1000 permutation test, a LOD threshold of 3.5 was set to find significant QTLs at the 95 % confidence level. The ranges above the LOD threshold of 3.5 were identified as QTL intervals. Markers located at or flanked with the peak LOD value of a QTL were recognized as QTL-associated markers.

### Candidate gene mining *in silico*

The corresponding regions of QTLs on the physical map were identified by mapping the associated markers. The genes within the QTL region, together with the functional annotations information, were available on the Genome Database for Rosaceae (GDR) website (http://www.rosaceae.org/species/malus/malus_x_domestica/genome_v1.0) [4]. Possible candidate genes related to a specific trait were predicted based on their biological functions. More attention was paid to functional genes, transcription factors, regulatory genes, and unknown genes.

### Validation of candidate genes related to fruit acidity

Because three QTLs involved in the segregation of fruit malic acid, citric acid, and total acid contents mapped to an overlapped genomic region on chromosome 08, six candidate genes, including three functional genes directly linked to fruit acidity, and three transcription factors were chosen for validation. Fruit of two cultivars, ‘Fuji’ and ‘Jonathan’, with low and high fruit acidity levels, respectively, were sampled at 30, 60, 90, 120, 150 (‘Jonathan’ ripening), and 175 (‘Fuji’ ripening) days after full bloom (DAFB). Each sample was collected from the flesh of six apples. Additionally, fruit acidity (content of malic acid, critic acid, and total acid) and gene expression were analyzed. Total RNA was extracted following the modified cetyltrimethylammonium bromide method described by Zhang et al. [38]. Extracts were digested using DNaseI (Takara, Dalian, China) at 37 °C for 30 min. After determining the concentration using a spectrophotometer (Nanodrop 2000; Thermo Scientific, Wilmington, DE, USA), 1 μg RNA of each sample was reverse transcribed into cDNA with the M-MLV kit (Takara, Dalian, China). Then, the gene expression was analyzed, using the cDNA samples, by an AB7500 Real-time PCR System and SYBR Green fluorescence dye (Takara, Dalian, China) [39].

The six analyzed candidate genes were MDP0000239624, MDP0000247324, MDP0000582174, MDP0000868410, MDP0000894463, and MDP0000599133. Among these genes, MDP0000247324 and MDP0000599133 failed to produce specific primers owing to their high homology with MDP0000894463 and MDP0000239624, respectively. The primers for the rest of the genes were designed using Primer Express 5.0 software (AuGCT, Beijing, China). Both β-actin and 18S-rRNA were analyzed as reference genes. Because of the high expression level of 18S-rRNA, only β-actin, which showed a similar expression level with our genes, was used to calculate the relative quantitative expression of genes. All primer sequences are listed in Additional file 1: Table S1.

## Results

### Marker development

Data from multiple Illumina/Solexa sequence channels was assorted by the appropriate 4–8 bp nucleotide multiplex identifier assigned to each sample. The raw data were modified by following two steps: first, the adapter and index sequences in reads were deleted, and then, the reads that contained more than 50 % low-quality bases (quality value ≤ 5) were removed. After filtering, the average Q20s of the samples were about 96 % (minimum 92.55 %), indicating the high quality of the data. Finally, 375.04 Gb of sequencing data were generated using *Eco*RI to digest, including 1.58 Gb of ‘Jonathan’, 1.80 Gb of ‘Golden Delicious’, and 371.66 Gb of the 318 F1 seedlings. The GC content of the parents was ~37 %, while the average of the hybrid population was 39.70 %. However, *Hin*dIII digestion produced 300.13 Gb of sequencing data, consisting of 2.10 Gb, 1.76 Gb, and 296.27 Gb of ‘Jonathan’, ‘Golden Delicious’, and the 318 hybrid seedlings, respectively. The GC content for both the parents and the progenies were almost 40 %. The average coverage levels of the F1 progeny were 1.57 (*Eco*RI) and 1.26 (*Hin*dIII). For ‘Jonathan’, the coverage levels were 2.13 (*Eco*RI) and 2.83 (*Hin*dIII), while they were 2.43 (*Eco*RI) and 2.38 (*Hin*dIII) for ‘Golden Delicious’ (Table 1).

**Table 1:** Quality evaluation of restriction-site associated DNA sequencing data in 318 hybrid seedlings and their parents

Restriction enzyme	Plant materials	Total reads (M)	Total bases (Gb)	GC (%)	Q20 (%)	Coverage (×)
*Eco*RI	Jonathan	17.12	1.58	37.33	97.04	~2.13
	Golden Delicious	19.04	1.80	37.15	96.95	~2.43
	Progeny	3972.27	371.66	39.70	96.16	~1.57
*Hin*dIII	Jonathan	22.33	2.10	39.32	96.78	~2.83
	Golden Delicious	18.77	1.76	39.26	96.79	~2.38
	Progeny	3164.47	296.27	39.37	96.86	~1.26

Data of progeny were collected from all 318 individuals derived from ‘Jonathan’ × ‘Golden Delicious’. The size of apple whole genome referenced the estimated genome size 742.3 Mb by Velasco et al. [4]

Based on the RAD tags, a high fidelity SNP dataset was generated. For each individual, two types of SNP were detected, one was the heterozygous loci itself, and the other was a homozygous loci but polymorphic amongst hybrid seedlings. Using *Eco*RI to digest, 15166 and 15255 SNPs were developed from ‘Jonathan’ and ‘Golden Delicious’, respectively. Among these SNPs, 9917 (‘Jonathan’, 65.39 %) and 9899 (‘Golden Delicious’, 64.89 %) were homozygous, while 5249 (‘Jonathan’, 34.61 %) and 5356 (‘Golden Delicious’, 35.11 %) were heterozygous loci. There were, in total, 14,094 SNPs detected amongst the hybrid seedlings, including 8151 (57.83 %) homozygous and 5943 (42.17 %) heterozygous loci. In contrast, 37,861 SNPs were obtained through *Hin*dIII digestion of ‘Jonathan’, including 28,531 homozygous and 9330 heterozygous loci, representing 75.36 % and 24.64 % of the total, respectively. In ‘Golden Delicious’, the total number of SNPs amounted to 37,742, 76.25 % of the total were homozygous and the rest, 23.75 %, were heterozygous loci. The number of SNPs generated from the progeny was 33,766 when subjected to *Hin*dIII digestion, more than twice the amount from *Eco*RI digestion, which indicated that the apple genome was rich in *Hin*dIII restriction sites. Meanwhile, the percentage of *Hin*dIII-digested homozygous SNP loci was higher, at 75.32 %, compared with heterozygous loci that only accounted for 24.68 % of the total (Table 2).

**Table 2:** Features of markers developed from restriction-site associated DNA sequencing in F1 progeny and parents

Restriction enzyme	Plant materials	No. of detected SNPs	No. of Homo loci /Homo rate (%)	No. of Hete loci /Hete rate (%)
*Eco*RI	Jonathan	15,166	9917/65.39	5249/34.61
	Golden Delicious	15,255	9899/64.89	5356/35.11
	Progeny	14,094	8151/57.83	5943/42.17
*Hin*dIII	Jonathan	37,861	28,531/75.36	9330/24.64
	Golden Delicious	37,742	28,780/76.25	8962/23.75
	Progeny	33,766	25,434/75.32	8332/24.68

The F1 progeny includes 318 individuals generated from ‘Jonathan’ × ‘Golden Delicious’. *Homo* homozygous, *Hete* heterozygous

### Map construction and marker alignment

SNP markers developed from RADseq cannot all be used in map construction. Because of the use of the F1 mapping population, homozygous markers with polymorphisms between the two parents were removed owing to non-segregation in the F1 progeny. High missing values affected the map orders and reduced the map accuracy [40]. To find appropriate missing values, groupings were calculated at different rates of missing data (10, 15, 20, and 25 %) with a LOD threshold of 6.0. With the 10 and 25 % missing values, the number of generated groupings was inconsistent with 17, the chromosome number of the diploid apple. However, we obtained 17 groupings with the 15 or 20 % missing values. There were 2312 valid markers with 15 % missing values and 3728 available markers with 20 % missing values, thus the 20 % missing value was chosen (Additional file 1: Table S2). Of the 3728 markers, 15 did not belong to a linkage group and, therefore, were removed. Furthermore, 42 markers were discarded after further analysis by the Chi-square test (p < 0.01). During the map construction, 230 markers were excluded because of their suspected or weak linkages with other markers, or their estimated positions changed.

During map construction, 21 seedlings were deleted from the mapping population, including 18 that showed several double recombination events and three with limited sequencing data, which caused more than 50 % missing genotyping data. Finally, the newly constructed consensus map contained 297 individuals and 3441 markers (1483 lm × ll, 1410 nn × np, and 548 hk × hk) (Additional file 2: Table S3). Markers mapped on each linkage group range from 121 to 334. The total length of the consensus map was 1650.2 cM with the average distance about 0.48 cM between markers. Using the ‘Create Maternal and Paternal Node’ function, maps of the two parents were constructed. Of the 3441 markers, 2017 were mapped to ‘Jonathan’ maps with a length of 1343.4 cM, and the average distance between markers was 0.67 cM; 1932 were mapped to ‘Golden Delicious’ maps with a length of 1516.0 cM, and the average distance between markers was 0.78 cM (Table 3; Additional file 3: Figure S1).

**Table 3:** Features of genetic linkage maps

Chromosome	Jonathan		Golden Delicious		Consensus	
/linkage group	No. of makers	Length(cM)	No. of makers	Length(cM)	No. of makers	Length(cM)
1	70	82.0	97	75.4	159	86.5
2	156	48.9	96	41.5	189	88.1
3	129	74.0	177	99.2	242	103.8
4	145	49.1	66	78.3	180	85.4
5	186	84.2	116	121.7	276	121.3
6	178	95.4	206	43.9	334	59.6
7	152	89.7	95	81.1	240	92.1
8	63	76.0	191	96.2	234	96.6
9	129	82.4	141	93.3	220	102.9
10	123	81.6	88	111.9	198	109.0
11	50	74.8	105	127.2	139	130.3
12	93	88.1	127	86.1	190	98.6
13	73	79.3	121	98.0	174	98.2
14	66	69.0	86	100.5	145	97.8
15	123	93.7	72	116.0	166	129.3
16	219	101.5	77	48.4	234	52.4
17	62	73.8	71	97.2	121	98.2
Total	2017	1343.4	1932	1516.0	3441	1650.2
Density (cM / maker)		0.67		0.78		0.48

The new maps were constructed using 3441 SNPs and 297 hybrid seedlings derived from ‘Jonathan’ × ‘Golden Delicious’. Marker amount and total length of each linkage group of parental and consensus maps were showed

**Fig. 1:**
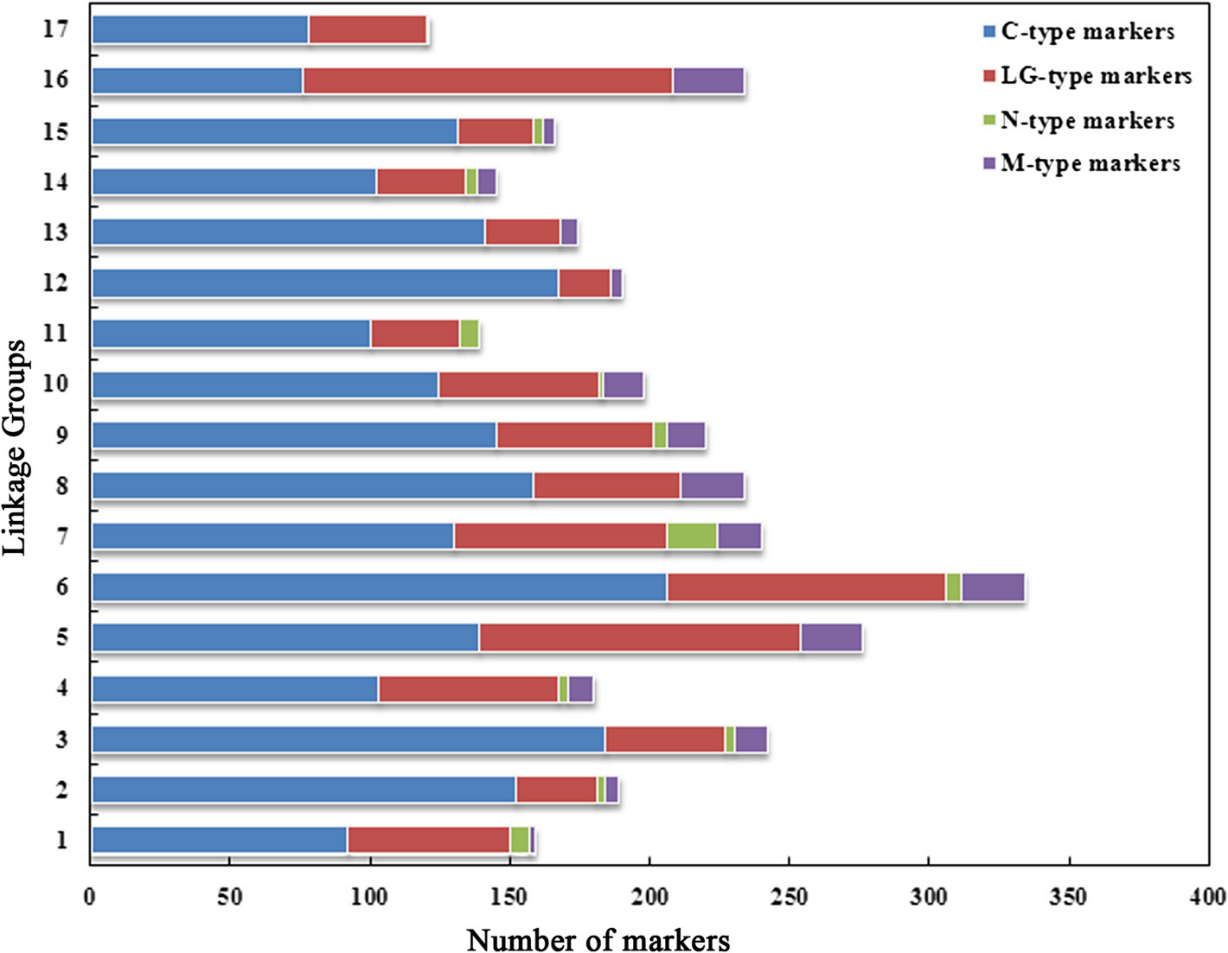
Distributions of different types of markers on each linkage group of consensus genetic maps. C-type markers: markers had consistent location of linkage map and reference genome of ‘Golden Delicious’. LG-type markers: markers had inconsistent location of linkage map and reference genome of ‘Golden Delicious’. M-type markers: markers aligned to unanchored chromosome of reference genome of ‘Golden Delicious’. N-type markers: markers cannot align to reference genome of ‘Golden Delicious’

All the markers on the genetic map were aligned to the reference genome of ‘Golden Delicious’ [4] and 98 % of that were successfully matched. There were 2228 C-type, 963 LG-type, and 189 M-type markers, respectively. However, there were 61 N-type markers that failed to match the current reference genome sequence. The distributions of four types of markers on each linkage group were calculated (Fig. 1). Among the 17 linkage groups, 132 LG-type markers, accounting for 56.41 %, were mapped on LG16, while there were only 19 LG-type markers (10.00 %) on LG12. LG-type markers from more than three different RAD tags that co-located on the linkage groups were defined as LG-type clusters. Markers within an LG-type cluster on LG01, LG05, LG06, LG09, and LG16, and two on LG06 of ‘Jonathan’ were mapped to the same chromosome, which deviated from their linkage groups. For example, the 20 SNP markers in the LG-type cluster located from 47.41 to 53.92 cM on LG09 of ‘Jonathan’ were almost mapped to the genomic region from 28.69 to 30.80 Mb on chromosome 13 of the reference genome. Four LG-type clusters were found in the linkage maps of ‘Golden Delicious’ (Table 4).

**Table 4:** Detail information of LG-type marker clusters on the genetic linkage maps

Cluster	Parent and linkage groups	Location on linkage maps (cM)	Number of makers	Anchored chromosome
1	J-LG01	19.16 ~ 19.40	5	Chr11
2	J-LG05	48.72 ~ 50.13	4	Chr10
3	J-LG06	27.54 ~ 27.66	3	Chr10
4	J-LG06	36.28 ~ 36.39	7	Chr14
5	J-LG09	47.41 ~ 53.92	20	Chr13
6	J-LG16	52.34 ~ 55.37	6	Chr04
7	G-LG01	15.53 ~ 15.63	5	Chr11
8	G-LG04	32.20 ~ 32.51	4	Chr06
9	G-LG05	88.51 ~ 90.74	6	Chr17
10	G-LG06	20.08 ~ 20.42	3	Chr10

*J* Jonathan, *G* Golden Delicious, and Chr: chromosome. The ‘anchored chromosome’ showed the uniquely aligned chromosome numbers of markers in an LG-cluster on the reference genome of ‘Golden Delicious’

### Frequency distributions of fruit quality traits

Fruit weight is a typical quantitative trait controlled by polygenes. Data on fruit weight were collected over four successive years from 2008 to 2011. The value of fruit weight varied continuously but had a non-normal distribution according to the Shapiro-Wilk test (Table 5; Additional file 4: Figure S2a-d). The population mean value of fruit weight was 128.77 ± 30.33 g (2008), 104.55 ± 23.67 g (2009), 110.94 ± 26.64 g (2010), and 81.30 ± 30.36 g (2011). The population mean value varied with year, indicating the significant impacts of environmental factors.

**Table 5:** An overview of population features of phenotyping data for each trait

Trait		Year	Maximum	Minimum	Mean(±SD)	Distribution
Fruit weight (g)		2008	253.80	56.80	128.77 (±30.33)	non normal
		2009	205.60	50.00	104.55 (±23.67)	non normal
		2010	193.60	52.00	110.94 (±26.64)	non normal
		2011	170.00	33.00	81.30 (±30.36)	non normal
Firmness (kg cm^−2^)		2010	15.67	3.84	8.78 (±1.81)	non normal
		2011	12.16	5.49	8.71 (±1.39)	normal
acidity	total acid	2011	11.547	2.118	6.887 (±2.285)	normal
(mg g^−1^)	malic acid	2011	11.211	1.653	6.452 (±2.322)	normal
	citric acid	2011	0.200	0.016	0.081 (±0.039)	normal
	tartaric acid	2011	0.310	0.010	0.109 (±0.056)	non normal
	oxalic acid	2011	0.099	0.048	0.070 (±0.011)	normal
	acetic acid	2011	1.021	0.000	0.169 (±0.214)	non normal
	succinic acid	2011	0.027	0.000	0.006 (±0.004)	non normal
sugar content	total sugar	2011	127.175	79.564	104.737 (±10.811)	normal
(mg g^−1^)	fructose	2011	68.477	33.302	51.014 (±6.854)	normal
	glucose	2011	35.065	9.239	19.690 (±6.176)	non normal
	sucrose	2011	61.584	11.059	34.033 (±9.765)	normal

Fruit weight were measured over 4 years, fruit firmness were determined over 2 years, whereas fruit acidity and sugar content were analyzed only in 2011. Each trait values were obtained from six fruits per n seedlings (*n* = 1170, 2008; *n* = 952, 2009; *n* = 527, 2010; *n* = 106; 2011). Distributions were calculated using Shapiro-Wilk test

Fruit firmness was measured in 2010 and 2011. The mean value was 8.78 ± 1.81 kg cm^−2^, while the minimum was 4.12 kg cm^−2^ and the maximum was 14.04 kg cm^−2^ in 2010. The average of fruit firmness in 2011 was 8.71 ± 1.39 kg cm^−2^, which approximately equaled the value in 2010. The data ranged from 5.49 to 12.16 kg cm^−2^. The frequency distribution of fruit firmness revealed a Gaussian distribution in 2011 (Table 5; Additional file 4: Figure S2e, f).

Fructose was the dominant soluble sugar in apple fruit. The content of fructose ranged from 33.302 to 68.477 mg g^−1^, with an average of 51.014 ± 6.854 mg g^−1^ in the population. The highest degree of variability was detected in fruit sucrose content (34.033 ± 9.765 mg g^−1^, 11.059–61.584 mg g^−1^). The fruit glucose content (19.690 ± 6.176 mg g^−1^, 9.239–35.065 mg g^−1^) was lower than fructose and sucrose contents across the population. Total sugar content (104.737 ± 10.811 mg g^−1^, 79.564–127.175 mg g^−1^) was derived by summing the contents of glucose, sucrose, and fructose. Based on the Shapiro-Wilk test, all of the sugar contents conformed to the law of normal distribution except for that of glucose (Table 5; Additional file 4: Figure S2g-j).

Fruit acidity was calculated based on the content of six types of organic acids. Malic acid, the main contributor of fruit acidity in apple, varied between 1.653 and 11.211 mg g^−1^, with a population mean value of 6.452 ± 2.322 mg g^−1^ and had a normal distribution. The contents of tartaric acid, citric acid, and oxalic acid were far lower than that of malic acid. Only the content of oxalic acid fit a normal distribution, while the contents of acetic acid and succinic acid showed biased distributions (Table 5; Additional file 4: Figure S2k-p).

### QTLs identified for fruit quality traits

A total of 12 significant QTLs were detected using the interval mapping method. Of the 12 QTLs, six contributed to fruit acidity, two were related to fruit sugar contents, two were associated with fruit firmness, and the remaining two were linked to fruit weight (Table 6). All these QTLs were confirmed by MQM mapping, and no additional QTLs that might be masked by other QTLs were identified (Additional file 5: Figure S3).

**Table 6:** QTLs for fruit quality traits detected by interval mapping

Trait	Year	QTL	Map/Linkage group	LOD	Exp (%)	Peak location (cM)	Intervals on maps (cM)	QTL-associated markers
Malic acid	2011	qtlma.j8	J8	7.7	39.1	57.27	55.27 ~ 59.15	emC08.14121899
								emC08.13666872
Citric acid	2011	qtlc.g8	G8	3.7	19.4	46.58	46.13 ~ 47.98	huM001766.113.1072
	2011	qtlc.j8	J8	5.5	27.2	59.15	55.27 ~ 62.15	emC08.14121899
	2011	qtlc.j15	J15	3.6	20.2	64.01	63.42 ~ 64.97	huC15.29186826
Acetic acid	2011	qtla.j7	J7	3.7	20.1	59.83	59.13 ~ 60.83	euLG07_014
Total acid	2011	qtlta.j8	J8	7.7	39.2	57.27	55.27 ~ 59.15	emC08.14121899
								emC08.13666872
Fructose	2011	qtlf.j1	J1	4.3	28.5	37.82	32.81 ~ 42.74	huC01.18233570
								emC01.11115376
Sucrose	2011	qtls.g1	G1	3.5	17.5	49.57	48.60 ~ 50.57	huC01.18378291
Fruit weight	2008	qtlfw.j3	J3	3.7	11.2	24.65	24.28 ~ 25.39	hmC03.5431144
	2008	qtlfw.j5	J5	3.8	11	53.86	52.86 ~ 54.86	euC05.25996313
Fruit firmness	2010	qtlff.j11a	J11	4.4	27.5	24.60	22.21 ~ 26.43	euC11.7408490
								huC11.10309880
	2010	qtlff.j11b	J11	3.9	21.6	11.48	10.48 ~ 13.30	euC11.5719084
								hmC11.4173064

The phenotyping data were collected from the mapping population derived from ‘Jonathan’ × ‘Golden Delicious’ in 2008 to 2011. QTL intervals were the ranges that above the LOD threshold of 3.5 at a 95 % confidence level. Markers located at or flanked with the peak LOD value of a QTL were recognized as QTL-associated markers. *J* Jonathan, *G* Golden Delicious

Of the six QTLs for fruit acidity, a major QTL (qtlma.j8) for malic acid, with a LOD score of 7.7 and a 39.1 % variance explanation, was mapped on LG08 of ‘Jonathan’. The markers emC08.14121899 and emC08.13666872 were associated with qtlma.j8. Three QTLs were identified as being linked to citric acid, one of them, qtlc.j8 (LOD = 5.5, 27.2 %), was identified near emC08.14121899 on LG08 of Jonathan. The second QTL for citric acid content was qtlc.j15 (LOD = 3.6, 20.2 %) on LG15 of ‘Jonathan’, associated with marker huC15.29186826. The third, qtlc.g8 with a LOD score of 3.7, was mapped to LG08 of ‘Golden Delicious’, which explained 19.4 % of the phenotypic variance. The marker huM001766.113.1072 was located at the peak of qtlc.g8. Additionally, qtlta.j8 (LOD = 7.7, 39.2 %), which was linked to total acidity, was detected on LG08 of ‘Jonathan’. Furthermore, qtla.j7 (LOD = 3.7, 20.1 %) on LG07 of ‘Jonathan’ was related to acetic acid. Among the QTLs for fruit acidity, qtlma.j8, qtlc.j8, and qtlta.j8 overlapped near the marker emC08.14121899 on LG08 of ‘Jonathan’, suggesting that this genomic region contained important genetic information related to fruit acidity. Based on the consensus map, huM001766.113.1072 associated with the QTL linked to citric acid on LG08 of ‘Golden Delicious’ was located 8.46 cM downstream of the marker emC08.14121899.

Two QTLs related to fruit sugar contents were detected. qtlf.j1 (LOD = 4.3, 28.8 %), which contributed to fruit fructose content, was mapped to LG01 of ‘Jonathan’, and the nearest marker was huC01.18233570. While qtls.g1 (LOD = 3.5, 17.5 %), which was linked to sucrose, was mapped to LG01 of ‘Golden Delicious’, and the nearest marker was huC01.18378291. The distance between the two markers, huC01.18233570 and huC01.18378291, was about 145 kb on the reference genome of apple.

In 2008, two QTLs of fruit weight were identified on genetic maps of ‘Jonathan’. One QTL, qtlfw.j3, was mapped on LG03, which explained 11.2 % of variance with a peak LOD score of 3.7. The other, qtlfw.j5, was mapped on LG05, with a LOD score of 3.8 and an 11.0 % explanation of variance.

There were two QTLs related to fruit firmness located on LG11 of ‘Jonathan’, qtlff.j11a (LOD = 4.4, 27.5 %) was associated with euC11.7408490 and huC11.10309880, while qtlff.j11b (LOD = 3.9, 21.6 %) was between marker hmC11.4173064 and euC11.5719084 (Table 6).

### Candidate genes involved in fruit quality traits

Based on the alignment of the QTL regions with the physical positions on the apple genome pseudo-chromosomes *in silico*, five QTLs with a region < 5 cM, having the highest LOD value and variance explanation among the three traits, had their corresponding positions identified, which were suitable for searching for candidate genes directly [41]. The five regions were 4.17-5.72 Mb of chromosome 11 (linked to fruit firmness), 7.40–10.31 Mb of chromosome 11 (linked to fruit firmness), 13.66-14.20 Mb of chromosome 08 (linked to malic, citric and total acid), 5.43–7.73 Mb of chromosome 03, and 22.64–26.00 Mb of chromosome 05 (both linked to fruit weight). Initially 366, 434, 194, 357, and 347 genes and their annotation within the five genomic regions, respectively, were obtained from the apple genome database. According to their functional predictions, 35, 29, 17, 35, and 45 genes, respectively, were good candidates (Additional file 6: Table S4).

### Analysis of fruit acidity and expressions of candidate genes

**Fig. 2:**
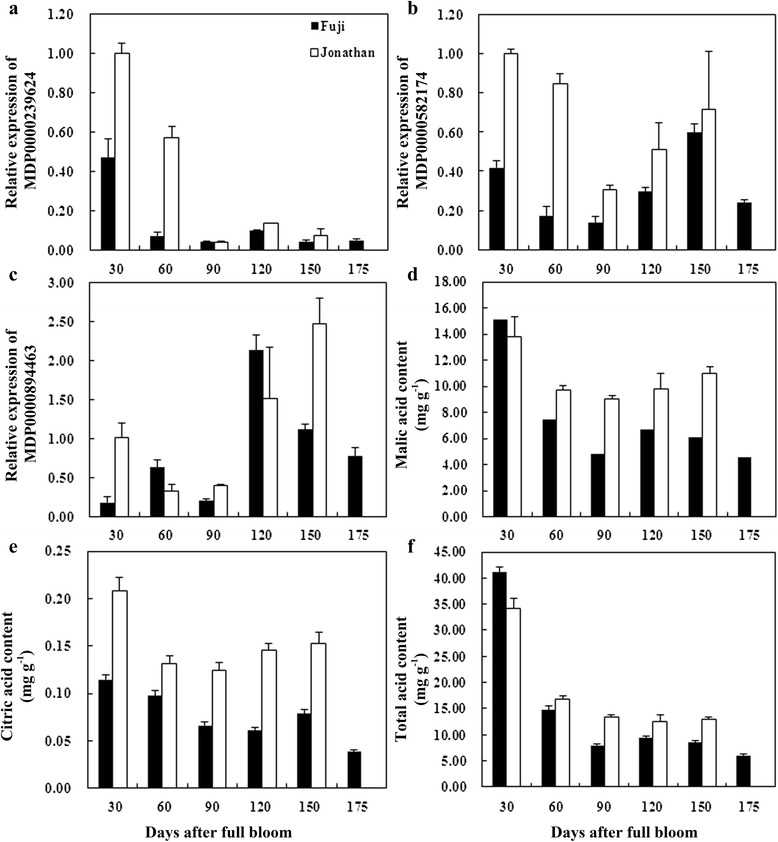
Changes of acid contents and expressions of candidate genes during fruit development in ‘Jonathan’ and ‘Fuji’. ‘Jonathan’ was chosen as a high acidity apple cultivar, while ‘Fuji’ as a low acidity cultivar. Expressions of three filtered candidate genes for fruit acidity were determined (showed in **a**-**c**). Since QTL on LG08 was linked to malic acid, citric acid and total acidity, **d**-**f** showed their dynamic changes during different periods of fruit development

**Fig. 3:**
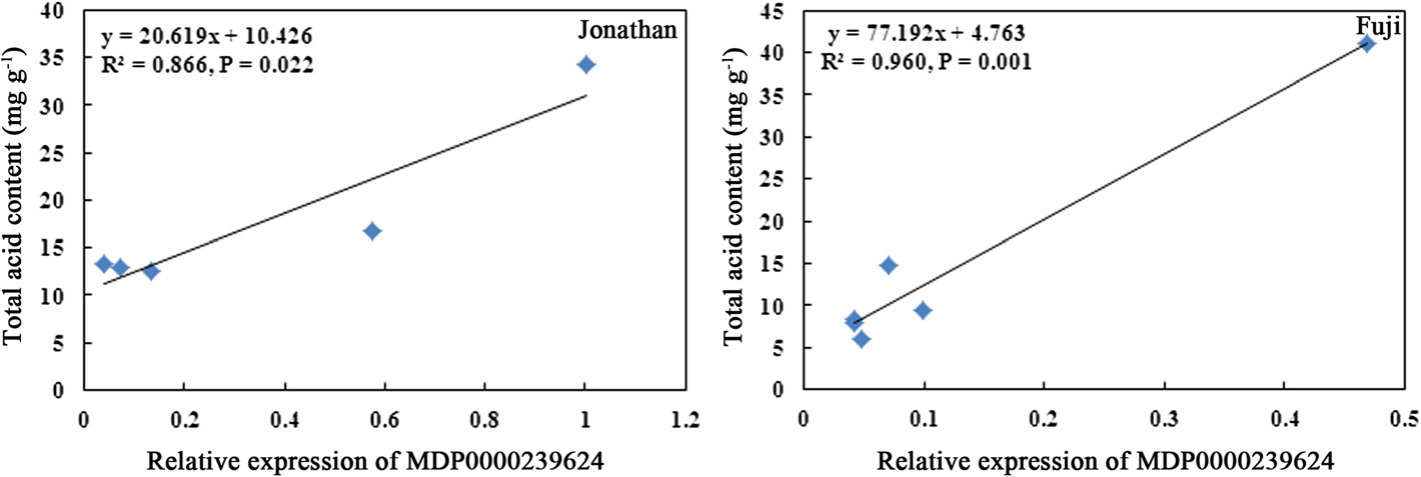
Regression between relative expression of MDP0000239624 and total acid content in ‘Jonathan’ and ‘Fuji’

To understand the potential relationship between candidate genes and fruit acidity, the expression profiles of candidate genes and fruit acidity were analyzed during different fruit development stages of a high acidity cultivar, ‘Jonathan’, and a low acidity cultivar, ‘Fuji’. The contents of malic, citric, and total acid were determined to indicate fruit acidity and the regularity of the data were confirmed. Generally, in young fruit of both ‘Jonathan’ and ‘Fuji’, high fruit acidity was detected at 30 DAFB, and then after 60 DAFB, the fruit acidity declined significantly. However, the acidity of ‘Jonathan’ was always higher than that of ‘Fuji’ (Fig. 2d-f). Among the analyzed genes, MDP0000868410 was not detectable in fruit tissue. MDP0000239624 (malic enzyme gene, *MdME*) expressed extensively in flesh tissue at 30 DAFB and 60 DAFB in both ‘Jonathan’ and ‘Fuji’, and the expression level was higher in ‘Jonathan’. After 90 DAFB, gene expression of MDP0000239624 was relatively low in both cultivars. The expression level of MDP0000582174 (*MdMYB4*) was reduced significantly during 60 DAFB to 90 DAFB and then, increased gradually from 120 DAFB to 150 DAFB in fruit of both ‘Jonathan’ and ‘Fuji’. The expression of MDP0000582174 was higher in ‘Jonathan’ than in ‘Fuji’ throughout the sampling seasons. The change pattern of expression level of MDP0000582174 was coordinate with that of fruit acidity. The expression of MDP0000894463 (*MdMYB44*) increased significantly from 120 DAFB to fruit ripening in fruit of both ‘Jonathan’ and ‘Fuji’ (Fig. 2a-c). Correlation analysis indicated that only the relative expression level of MDP0000239624 was significantly correlated with the total acid content (*R*^*2*^ = 0.866, *P* = 0.022 in ‘Jonahan’; *R*^*2*^ = 0.960, *P* = 0.001 in ‘Fuji’) (Fig. 3). These results suggested that transcription factor *MdMYB4* and functional gene *MdME* may be involved in the segregation of fruit acidity.

## Discussion

This was the first time that large numbers of SNP markers were developed using RADseq in apple. For a species with high heterozygocity, more than one restriction enzyme may be required to reveal more molecular diversity [42]. In this work, a total of 842 valid SNP markers were generated through *Eco*RI digestion for map construction. While the three times more SNPs, 2599, were detected via *Hin*dIII digestion. Besides RADseq, SNP markers developed using expressed sequenced tags (ESTs) database, BAC-end sequences, and chips have been reported in apple [43–46]. By comparison with RADseq, EST-based SNPs have low coverage of the genome but useful for bridging functional and structural genomics [43]. BAC-end sequence-based SNPs were also limited in marker abundance and depended on BAC library which was not easy to construct [44]. SNP arrays and chips needed genomic information in advance and were always custom designed or commercialized, which means it was costly for screening a big population [45–47]. Altogather, it is suggested that RADseq was a rapid, high efficient and less cost method for molecular breeding using larger population and larger amount of markers.

In total, 963 markers approach to 30 percent of mapped markers were mapped to unexpected positions when aligned to the apple genome sequence. This was due at least in part to non-unique sequences aligning by partial homology between non-homologous chromosomes in the apple genome, such as chromosome 1/7, 2/15/8, 3/11, 4/12, 5/10, 6/14, 9/17, and 13/16 [4, 48]. For example, marker huLG03_029 was mapped to LG03 according to its linkage relationship to the neighboring markers, but the best sequence match in the genome was on chromosome 11. This type of event had been reported previously. The primer sequences of SSR marker Hi04a05 aligned to chromosome 01, but it mapped to LG09 in the ‘Fiesta’ genetic map, while NZmsEB177464.z mapped to LG03 of the M432 map, contrary to the best sequence match on chromosome 02 of the ‘Golden Delicious’ [49, 50]. Another possible reason for mapping markers on unexpected linkage groups was chromosome structural variations, which has also been reported in the apple genome [4].

Some clustered LG-type markers on genetic maps reminded us there may be some chromosome fragment rearrangement events occurred in both parents. In the region from 47.41 to 53.92 cM of LG09 of the ‘Jonathan’ map, in total of 20 SNP makers, including huLG09_042, huLG09_044, and huLG09_018 *etc*., were uniquely matched to chromosome 13. This implied a probability of a large chromosome fragment rearrangement between chromosome 09 and 13 in ‘Jonathan’ genome. Additionally, other nine regions were noted in both parents with 3 to 7 markers (Table 4). Similarly, in *Capsicum*, a chromosomal translocation event between chromosome 01 and 08, between wild and cultivated *Capsicum*, respectively, had been reported based on an EST-based linkage map [51]. The validation and biological significance of chromosome structural variations are often analyzed using fluorescence in situ hybridization [51, 52], and we have already been working on it and the results could be expected soon.

The QTLs for fruit acidity detected on LG08 or chromosome 08 were suggested that there was a major gene affecting fruit acidity in this region. In our previous study using an SSR genetic linkage map of the same population ‘Jonathan’ × ‘Golden Delicious’, a QTL for malic acid was mapped on LG08 associated with the marker CH05a02y explaining 13.5 % of population variance [23]. Because of the low density of the SSR genetic map, the QTL interval was about 23.60 cM. Here, the QTLs qtlma.j8, qtlc.j8, and qtlta.j8 linked to citric acid, malic acid, and total acidity, respectively, were mapped to a similar genomic region on LG08 of ‘Jonathan’, with the highest LOD score of 7.7 and more than 39 % variance explanation. The QTL interval was around 4 cM for malic acid, and the marker emC08.14121899, nearest to qtlma.j8, was ~2.3 Mb apart from CH05a02y on the apple genome [23]. In addition, a QTL on LG08 for fruit acidity had also been detected near the AFLP marker E31M38-0193 using the population ‘Fiesta’ × ‘Discovery’ [21].

It is known that the regulation of apple fruit acidity involves several key enzymes, such as MdPEPC, MdcyMDH, and MdcyME [53, 54]. Based on the results of genomic selection for fruit acidity in apple, the SNP marker ss475882883 on LG08 (19658610) had the largest effect was co-localized with a RING finger and CHY zinc finger domain-containing protein gene (MDP0000294924) [28]. In our QTL region on chromosome 08, two genes encoding NADP-dependent malic enzyme were found. The malic enzyme could influence the malic acid content by catalysing the conversion of malic acid to pyruvic acid [53]. Candidate genes for fruit acidity also included three MYB transcription factors and a citrate-binding protein.

QTL for total soluble solids in apple had never been mapped to LG01 before, however, a QTL on LG01 was detected recently by gas chromatography–mass spectrometry for sugar composition [30]. Two major QTLs, qtlf.j1 and qtls.g1, linked to fruit sugar contents were identified on LG01 in this paper by HPLC analysis. The distance between markers huC01.18233570 and huC01.18378291 associated with qtlf.j1 and qtls.g1, respectively, was about 145 kb on the genome sequence.

Two QTLs, qtlff.j11a and qtlff.11b, for fruit firmness were mapped to LG11. In ‘Fiesta’ × ‘Discovery’, 10 years ago, a QTL for fruit firmness was also mapped on LG11 despite that fact that the linked AFLP markers cannot be anchored to the genome [21]. Later, using a genomic selection approach, one of the three largest SNP effects on fruit firmness was also located on LG11, but no known candidate genes were found at this genomic region [28]. During fruit ripening, changes in ethylene and the cell wall structure affected fruit firmness, therefore the genes involved in cell wall composition, modification, ethylene metabolic or signaling pathways were considered to be candidates [32, 55–58]. In the QTL regions of qtlff.j11a and qtlff.11b, 64 genes were potential candidates for fruit firmness.

Several QTLs related to fruit weight were detected on LG03 and LG05. In the ‘Co-op17’ × ‘Co-op16’ population, QTLs for fruit weight on LG03 and LG05 were closely linked to markers CTG1069342 and CH01b07, with QTL intervals extended over 20.34 and 26.26 cM, respectively [59]. Using the same population ‘Jonathan’ × ‘Golden Delicious’ but an SSR genetic map, two minor QTLs related to fruit weight were mapped on LG05 of ‘Jonathan’ map, flanked with marker Hi02a03 and LG03 of ‘Golden Delicious’ map, linked to marker WBGCAS27 [60]. Because of the incompatible physical and genetic positions of Hi02a03 and WBGCAS27 [23], the genomic regions of these two QTLs could not be estimated accurately. Combined the results of predecessors with our experiment, hmC03.5431144 associated with qtlfw.j3 for fruit weight on LG03, was on the opposite end from the location of CTG1069342, and euC05.25996313 linked to qtlfw.j5, was about 7 Mb away from CH01b07 on the apple genome. The QTL intervals were narrowed down apparently to 1.11 cM for qtlfw.j3 and 2 cM for qtlfw.j5. Fruit weight is facilitated by cell division at the early growth stage and cell expansion during the later growth stage. According to previous research, some genes, such as E3 ubiquitin ligase regulating cell proliferation, CDKB2 controlling cell division, and auxin response factors *etc*. may be potential candidates [31, 61–63].

Summarized the QTL mapping results in our study, we found that for traits such as fruit firmness and fruit weight, QTLs were detected in only one of the successive years of measurement in this study. This may be because that the minor QTL loci are not year-stably detected except for in a larger population [64]. Moreover, environmental factors have a marked influence on fruit weight, which could have led to differences in QTL mapping results [65]. However, we have combined our obtained QTLs for fruit quality traits with previously reported QTLs and most of them showed a good agreement. Meanwhile candidate genes were searched in QTL regions. All these results could confirm that the QTLs identified in this study were reliable.

Candidate genes searching based on QTL regions was universal in current breeding programs [66–68]. It is suggested that candidate genes could perform an important role in molecular breeding. On one hand, functional genes related to a specific trait could be transformed as DNA markers using in QTL analysis and MAS. For example, *PG1* and *ACO1* were tightly linked to QTLs mapped on LG10 for fruit firmness in apple [32, 33, 67]; *LAR1* was associated with QTL cluster located on LG16 for polyphenolic composition in apple fruit [69]. A mutation, transition G to A, at base 1455 in the open reading frame of an *ALMT* (Aluminum-activated malate transporter) gene leads to a premature stop codon is responsible for the low acidity of apple fruit [68], which could be used in earlier stage selection of breeding process. On the other hand, the expression level of trait related genes also can help for selection. Assisted by the expression levels of *MxHA7*, *MxFIT1*, *MxIRT1*, *MxCS1* and *MxRD3*, 14 iron deficiency tolerant lines were preliminarily selected out of the 141 hybrids between *Malus xiaojinensis* and *M. baccata* [70]. In our manuscript, candidate genes were searched in QTL regions related to three fruit quality traits. The expression level of functional genes and transcription factors obtained in QTL on LG08 for fruit acidity were analyzed. The results showed that changes in the expression of *MdMYB4* (MDP0000582174) were in agreement with the pattern of malic acid content in apple, and the relative expression of *MdME* (MDP0000239624) was significantly correlated with fruit acidity, suggesting their possible involvement in affecting fruit acidity. In Summary, it is supposed that the dedicated studies of candidate genes could provide a new insight into the genetic control of fruit quality traits, and help the further research of QTLs that were not year stable. Now, studies of the other acquired candidate genes in this paper are still in progress. The applications of our candidate genes in future breeding projects could be expected.

## Conclusion

This study provides insights into map construction and map based molecular breeding method in apple. RADseq, first time performed on apple, was a rapid and efficient strategy for SNP maker development and genotyping in big mapping populations. A main contribution was that we constructed a new standard genetic map of apple with 3441 SNP markers and 297 individuals derived from a cross between ‘Jonathan’ and ‘Golden Delicious’. This may improves the accuracy of genetic maps greatly. Based on the map, 12 reliable QTLs responsible for fruit quality traits were detected. These QTL regions were narrowed down substantially and easy to find their corresponding positions on the genome sequence due to the associated markers. Subsequently, candidate genes were effectively predicted in the QTL regions less than 5 cM, and some of them were validated using real time PCR.

Briefly, a high-density genetic linkage map was generated in apple. The availability of such maps may facilitate a variety of genomic studies in apple, including QTL analysis, candidate genes searching and chromosomal variation.

### Availability of supporting data

The data sets supporting the results of this article are included within the article and its additional files.

## Additional files

Additional file 1: 
**Table S1.** Primer sequences for candidate genes analyzed by real-time PCR.
** Table S2.** Numbers of linkage groups and estimated markers by different rates of missing data with LOD = 6.0. (PDF 116 kb) Additional file 2: Table S3. List of genotyping data of 3441 SNP markers among 297 F1 seedlings. (XLSX 4309 kb) Additional file 3: Figure S1. Genetic linkage maps generated using 297 hybrid seedlings derived from ‘Jonathan’ × ‘Golden Delicious’. The 17 linkage groups represented the 17 chromosomes of *Malus* × *domestica*. The common markers between each parental map and consensus map were showed by the read lines. LG: Linkage groups; J: Jonathan; G: Golden Delicious. (RAR 19136 kb) Additional file 4: Figure S2. Frequency distribution diagrams of fruit quality traits in an F1 population derived from ‘Jonathan’ × ‘Golden Delicious’. Fruit weight were measured from year 2008 to 2011 showed in a-d, fruit firmness were determined in 2010 and 2011 showed in e and f, whereas sugar content consist of three composition (g-j) and fruit acidity including six kinds of organic acid and total acidity (k-p) were analyzed only in 2011. Each trait values were obtained from six fruits per n seedlings (*n* = 1170, 2008; *n* = 952, 2009; *n* = 527, 2010; *n* = 106; 2011). (TIFF 3001 kb) Additional file 5: Figure S3. QTLs for fruit quality traits identified by multiple QTL mapping (MQM). The results were consistent with that obtained by interval mapping. J: Jonathan, G: Golden Delicious. (TIFF 461 kb) Additional file 6: Table S4. Candidate genes searched from the regions of five significant QTLs (interval < 5 cM). These five QTLs were associated with three important fruit quality traits, two of fruit firmness, two of fruit weight and one of fruit acidity. The gene function annotation information of whole apple genome was downloaded from GDR website (https://www.rosaceae.org/species/malus/malus_x_domestica/genome_v1.0). Candidate genes which have been used for expression analysis were in bold fonts. (XLSX 20 kb)
