# Supplementary material for: A dense SNP genetic map constructed using restriction site-associated DNA sequencing enables detection of QTLs controlling apple fruit quality
Source: BMC Genomics. 2015 Oct 5;16:747. doi: 10.1186/s12864-015-1946-x (PMC4595315; doi:10.1186/s12864-015-1946-x)
Supplement: Additional file 1: — Table S1. Primer sequences for candidate genes analyzed by real-time PCR. Table S2. Numbers of linkage groups and estimated markers by different rates of missing data with LOD = 6.0. (PDF 116 kb) [file 12864_2015_1946_MOESM1_ESM.pdf]

Additional files

A dense SNP genetic map constructed using restriction site-associated DNA sequencing enables detection of QTLs controlling apple fruit quality

Rui Sun[1], Yuansheng Chang[1], Fengqiu Yang[2], Yi Wang[1], Hui Li[1], Yongbo Zhao[2], Dongmei Chen[2], Ting Wu[1], Xinzhong Zhang[1,*], Zhenhai Han[1,*]

1 Institute for Horticultural Plants, College of Agronomy and Biotechnology, China Agricultural University, Beijing 100193, China

2 Changli Institute for Pomology, Hebei Academy of Agricultural and Forestry Science, Changli, Heibei 066600, China

[*] Corresponding author

Xinzhong Zhang[1,*] and Zhenhai Han[1,*]

Email: zhangxinzhong999@126.com; rschan@cau.edu.cn

Telephone: +86 10 62734391

Fax: +86 10 62734391

Additional file 1: additional file 1.pdf

Table S1. Primer sequences for candidate genes analyzed by real-time PCR.

Gene ID	Primer sequences	
MDP0000894463	Fwd	5'-TGTGCTCGTCATCTACTTCGTCTT-3'
	Rev	5'-AAGATGGTCTCGTCCTCGGC-3'
MDP0000239624	Fwd	5'-GGAGCGTAGTGGAGGAGATA-3'
	Rev	5'-GTTAGGACGGTTGGAGGC-3'
MDP0000582174	Fwd	5'-TGTGAAAAGGCTCACACCAACAAAG-3'
	Rev	5'-TTGCCATGATTGAGATCAGGTCTAAGGT-3'
MDP0000868410	Fwd	5'-TTGTGCCAAAATGGAACCTCT-3'
	Rev	5'-TCTGGACTCCGTCAATGAAAATA-3'
β-Actin	Fwd	5'-TGGTGAGGCTCTATTCCAAC-3'
	Rev	5'-TGGCATATACTCTGGAGGCT-3'
18S-rRNA	Fwd	5'-CCTCCAATGGATCCTCGTTA-3'
	Rev	5'-ACACGGGGAGGTAGTGACAA-3'

Table S2. Numbers of linkage groups and estimated markers by different rates of missing data with LOD=6.0.

Rate of missing data (%)	No. of linkage groups	No. of markers
10	19	898
15	17	2312
20	17	3728
25	16	5034

Additional file 2: Table S3.xlsx

Table S3. List of genotyping data of 3441 SNP markers among 297 F1 seedlings.

Additional file 3: Figure S1.rar (figures in the zip file were .tif format)

Figure S1. Genetic linkage maps generated using 297 hybrid seedlings derived from 'Jonathan' × 'Golden Delicious'. The 17 linkage groups represented the 17 chromosomes of Malus × domestica. The common markers between each parental map and consensus map were showed by the read lines. LG: Linkage groups; J: Jonathan; G: Golden Delicious.

Additional file 4: Figure S2.tif

Figure S2. Frequency distribution diagrams of fruit quality traits in an F1 population derived from 'Jonathan' × 'Golden Delicious'. Fruit weight were measured from year 2008 to 2011 showed in a-d, fruit firmness were determined in 2010 and 2011 showed in e and f, whereas sugar content consist of three composition (g-j) and fruit acidity including six kinds of organic acid and total acidity (k-p) were analyzed only in 2011. Each trait values were obtained from six fruits per n seedlings (n=1170, 2008; n=952, 2009; n=527, 2010; n=106; 2011).

Additional file 5: Figure S3.tif

Figure S3. QTLs for fruit quality traits identified by multiple QTL mapping (MQM). The results were consistent with that obtained by interval mapping. J: Jonathan, G: Golden Delicious.

Additional file 6: Table S4.xlsx

Table S4. Candidate genes searched from the regions of five significant QTLs (interval < 5 cM). These five QTLs were associated with three important fruit quality traits, two of fruit firmness, two of fruit weight and one of fruit acidity. The gene function annotation information of whole apple genome was downloaded from GDR website (https://www.rosaceae.org/species/malus/malus_x_domestica/genome_v1.0). Candidate genes which have been used for expression analysis were in bold fonts.
