# Supplementary figures and images for: A dense SNP genetic map constructed using restriction site-associated DNA sequencing enables detection of QTLs controlling apple fruit quality
Source: BMC Genomics. 2015 Oct 5;16:747. doi: 10.1186/s12864-015-1946-x (PMC4595315; doi:10.1186/s12864-015-1946-x)

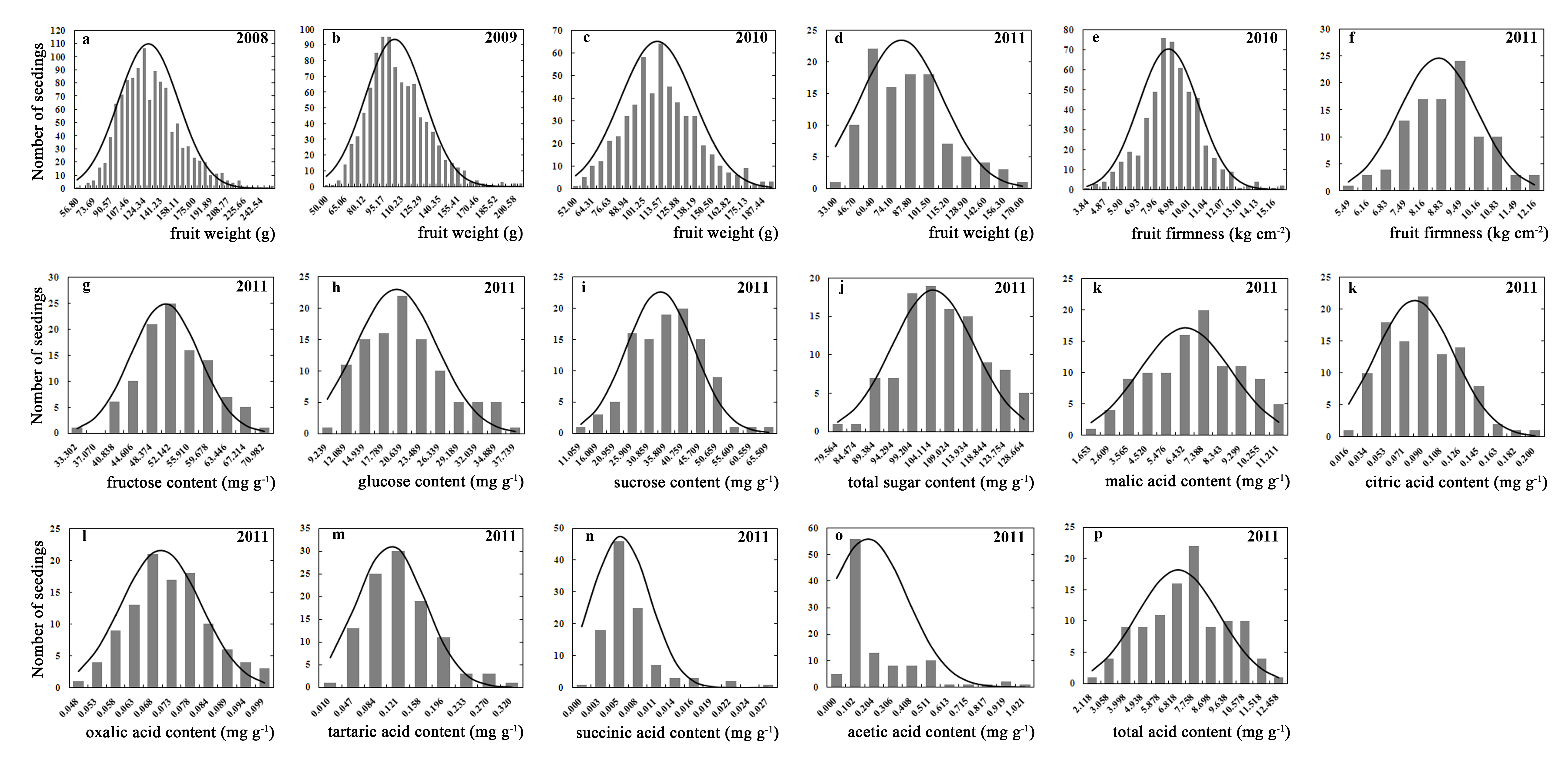

Supplement: Additional file 4: Figure S2. — Frequency distribution diagrams of fruit quality traits in an F1 population derived from ‘Jonathan’ × ‘Golden Delicious’. Fruit weight were measured from year 2008 to 2011 showed in a-d, fruit firmness were determined in 2010 and 2011 showed in e and f, whereas sugar content consist of three composition (g-j) and fruit acidity including six kinds of organic acid and total acidity (k-p) were analyzed only in 2011. Each trait values were obtained from six fruits per n seedlings (n = 1170, 2008; n = 952, 2009; n = 527, 2010; n = 106; 2011). (TIFF 3001 kb) [file 12864_2015_1946_MOESM4_ESM.tiff]

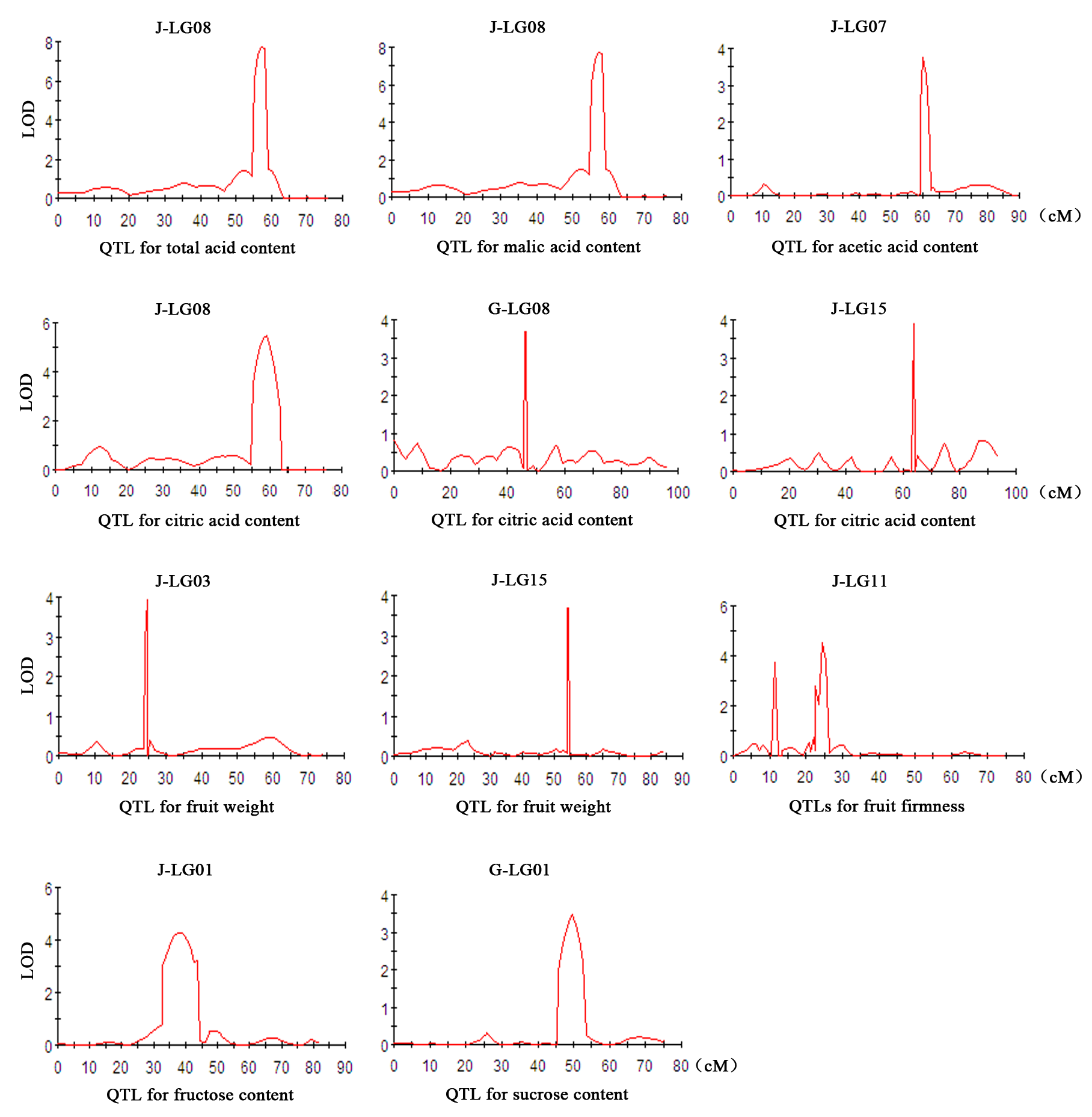

Supplement: Additional file 5: Figure S3. — QTLs for fruit quality traits identified by multiple QTL mapping (MQM). The results were consistent with that obtained by interval mapping. J: Jonathan, G: Golden Delicious. (TIFF 461 kb) [file 12864_2015_1946_MOESM5_ESM.tiff]
